# Supplementary material for: Prenatal thalamic waves regulate cortical area size prior to sensory processing
Source: Nat Commun. 2017 Feb 3;8:14172. doi: 10.1038/ncomms14172 (PMC5296753; doi:10.1038/ncomms14172)
Supplement: Supplementary Information — Supplementary Figures. [file ncomms14172-s1.pdf]

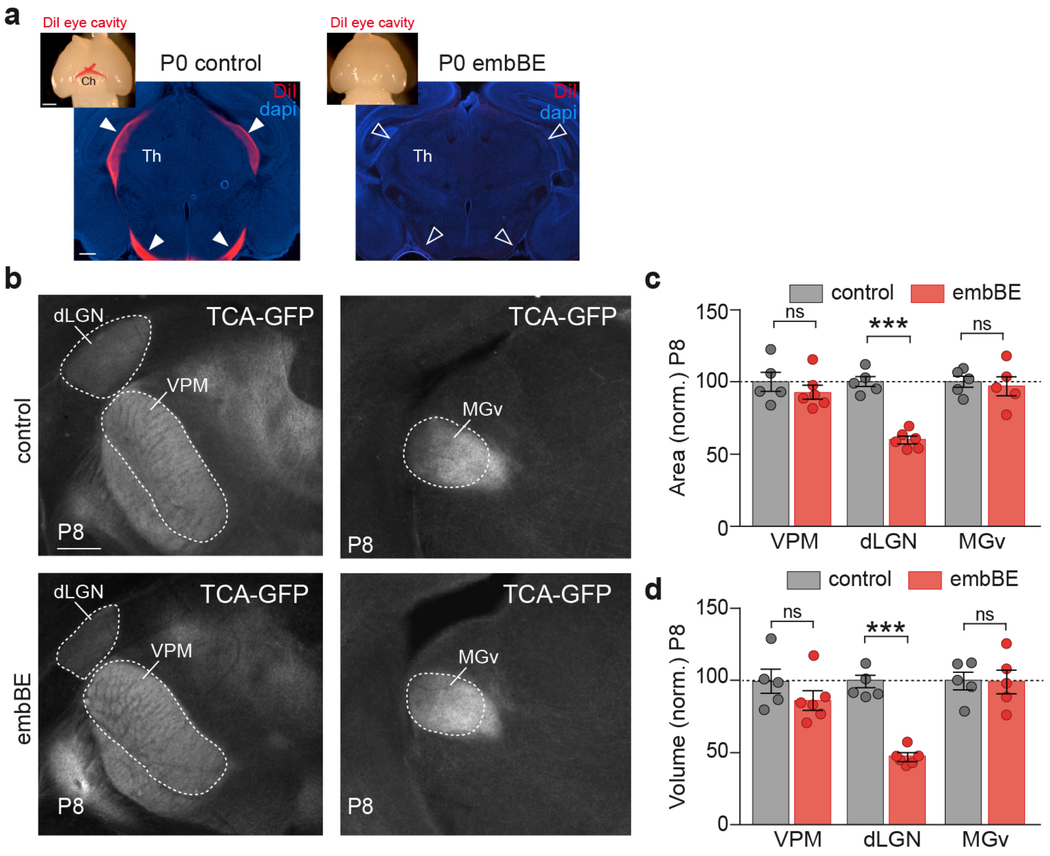

Supplementary Figure 1 Embryonic complete ablation of the retinal input (embBE) triggers a reduction in the dLGN size at P8. (**a**) Bilateral DiI injections into the eye cavity of control and embBE mice at P0 revealing the efficiency of the *in utero* enucleation. (**b**) Coronal sections from the TCA-GFP transgenic mice showing the area of the dLGN, VPM and MGv nuclei in control and embBE conditions. (**c**) Quantification of the area size for each principal thalamic nucleus shown in **b** (dLGN: control: 100 ± 3.61%, n = 5; embBE: 60.18 ± 2.46%, n = 6 ***P < 0.001. VPM: control: 100 ± 6.54%, n = 5; embBE: 92.84 ± 4.89, n = 6; P = 0.39. MGv: control: 100 ± 3.76%, n = 6; embBE: 97.29 ± 6.63%, n = 5; P = 0.73.; Two-tailed Student's t-test). (**d**) Quantification of the dLGN, VPM and MGv volume at P8 (dLGN: control: 100 ± 4.48%, n = 5; embBE: 46.96 ± 2.35% n = 6; ***P <

0.001. VPM: control: 100 ± 8.49%, n = 5; embBE: 86.46 ± 6.85, n = 6; P = 0.24. MGv: control: 100 ± 6.32%; embBE: 99.8 ± 8.55%; P = 0.98; Two-tailed Student's t-test). Graphs represent mean ± SEM. Scale bars, 300μm (1mm, insets in **a**).

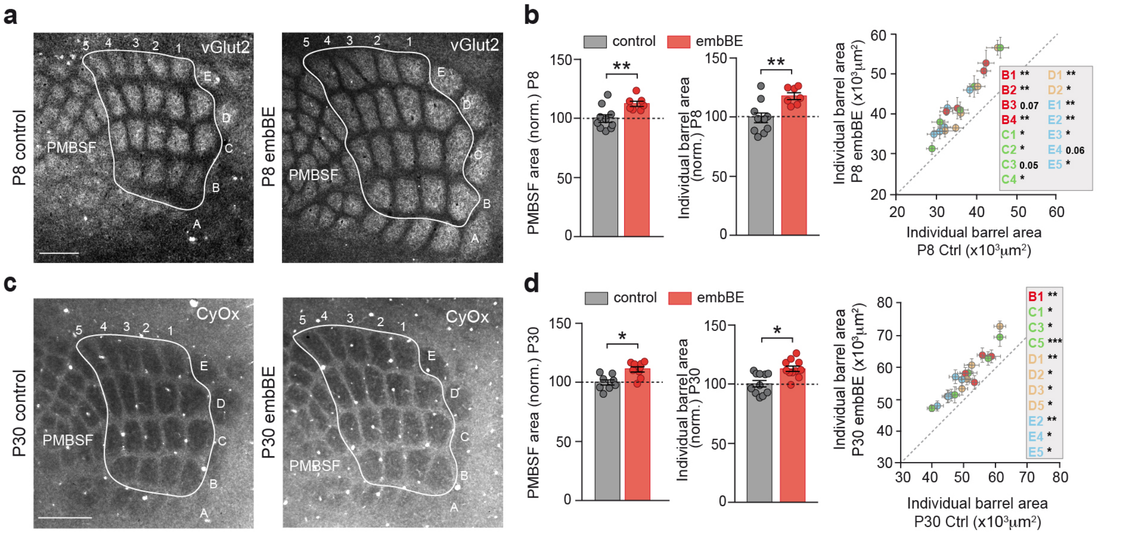

Supplementary Figure 2 EmbBE triggers experience-independent adaptations in S1 at P8 that are maintained in the adult. (**a**) vGlut2-immunostaining of the PMBSF in the S1 of control (*n* = 10) and embBE (*n* = 7) mice at P8. (**b**) Quantification of the total PMBSF area and the individual barrel area in the control and embBE brains at P8 (control: 100 ± 3.11%, *n* = 10; embBE: 112.4 ± 2.27%, *n* = 7; $^{**}P = 0.01$; control: 100 ± 4.27%, *n* = 10; embBE: 118.7 ± 2.74%, *n* = 7; $^{**}P = 0.016$, respectively; Two-tailed Student's t-test). Right panel: plot of each individual barrel area at P8. (**c**) CyOx (Cytochrome Oxidase) staining of the PMBSF in the S1 of control and embBE mice at P30. (**d**) Quantification of the total PMBSF area (control: 100 ± 2.2%, *n* = 8; embBE: 111.2 ± 2.1%, *n* = 9, $^{**}P = 0.0026$; Two-tailed Student's t-test) and individual barrel area (control *n* = 11; embBE *n* = 11 $^{**}P = 0.0018$; Two-tailed Student's t-test). Right panel: plot of each individual barrel area at P30. Graphs represent mean ± SEM. Scale bars, 300µm in **a**, and 500µm in **c**.

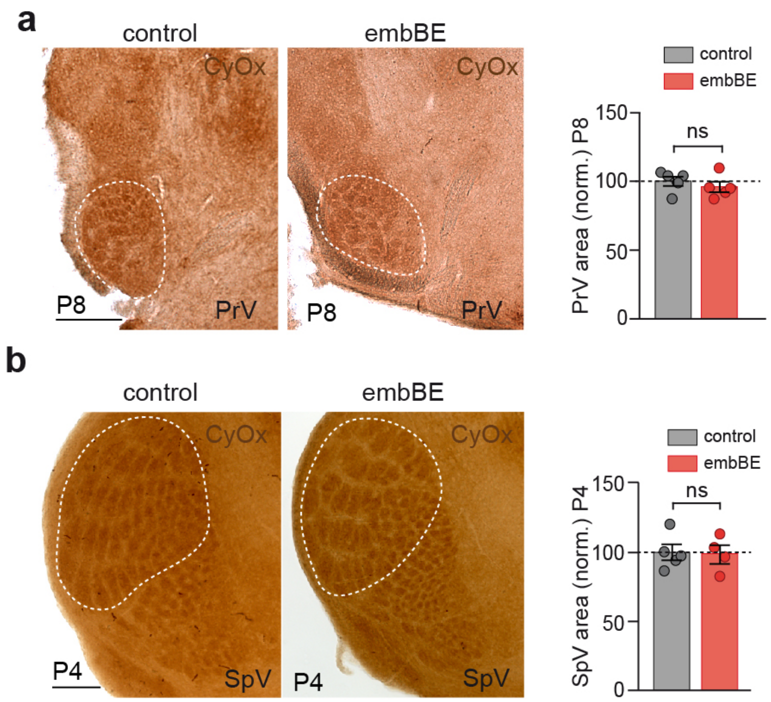

Supplementary Figure 3 Subcortical somatosensory structures are not affected in embBE mice. (**a**) CyOx staining showing the barrelettes in the PrV nucleus of the hindbrain in control and embBE animals at P8, quantification of the total PrV area (control 100 ± 3.36%, $n = 5$; embBE: 95.8 ± 3.76%, $n = 5$; $P = 0.42$; Mann-Whitney U-Test). (**b**) CyOx staining showing the barrelettes in the SpV nucleus of the hindbrain in control and embBE animals at P8, quantification of the total SpV area (control 100 ± 5.76%, $n = 5$; embBE: 98.6 ± 6.5%, $n = 4$; $P>0.99$; Mann-Whitney U-Test). Graphs represent mean ± SEM. Scale bars, 300µm.

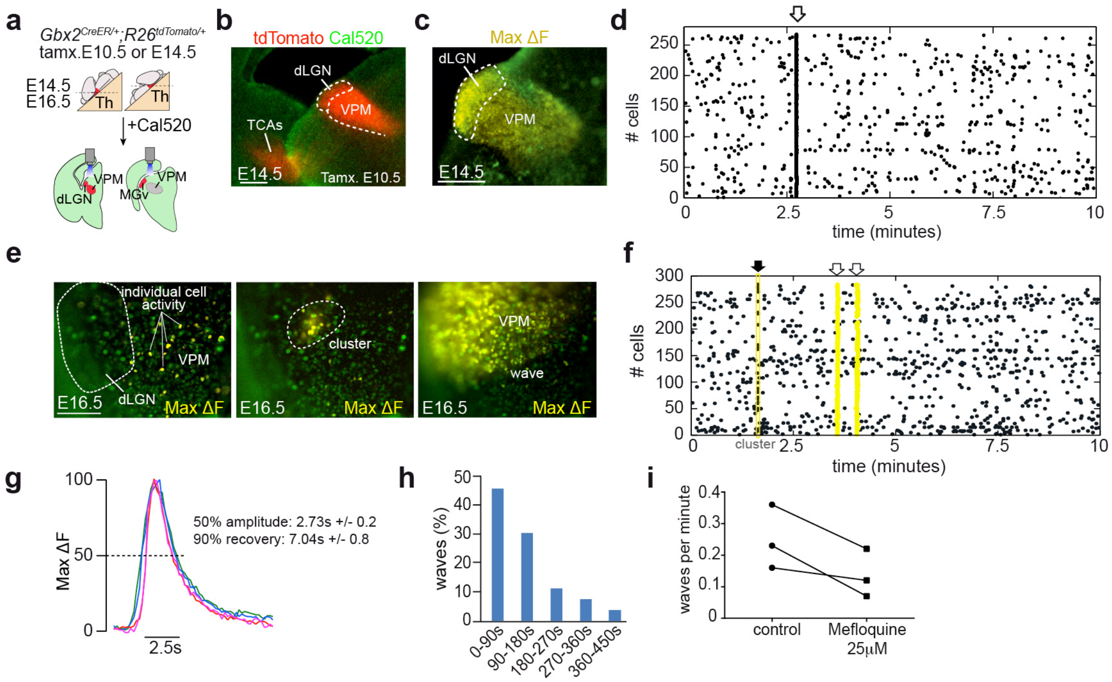

Supplementary Figure 4 Spontaneous Ca^{2+} activity in the developing thalamus. (**a**) Squematic representation of the different orientations used to obtain the thalamocortical acute slices. Embryonic acute thalamocortical slices from *Gbx2*$^{CreER/+}$*;R26*$^{tdTomato/+}$ mice with tamoxifen administrated at E10.5 (to label dLGN and VPM) or at E14.5 (to specifically label MGv). (**b**) Acute slice showing tdTomato in the dLGN and VPM nuclei and loaded with Cal 520^{TM} calcium dye at E14.5. Thalamocortical projections (TCAs) are also labeled. (**c**) Maximum projection of a Ca^{2+} wave (yellow) at E14.5 covering the dLGN-VPM nuclei. (**d**) Raster plot showing the onsets of individual cells Ca^{2+} transients between dLGN-VPM nuclei at E14.5. Open arrow indicates a wave. (**e**) Asynchronous Ca^{2+} transients in individual thalamic cells (left), clusters of synchronously co-active thalamic cells (middle) and a Ca^{2+} wave (right) at E16.5. (**f**) Raster plot showing the onsets of individual cells Ca^{2+} transients. Clusters are represented by small groups of cells with

simultaneous Ca^{2+} transients onsets (black arrow), while the waves involve almost all the cells within the nucleus (open arrows), including those that remain predominantly inactive during the inter-wave intervals. (**g**) Kinetics of the Ca^{2+} transients during a wave in four different ROIs. The amplitude of the transients has been normalized and the time aligned in the maximum. Wave width at 50% of the maximum amplitude: 2.73±0.2s and 90% recovery: 7.04±0.8s after wave onset. (**h**) Relative frequency distribution of inter-wave intervals measured at 90s periods. (**i**) Quantification of the effect on the frequency of dLGN-VPM waves after treatment with the connexin36 gap junction blocker, Mefloquine (25µM). Scale bars, 200µm in **b** and **c**, and 100µm in **e**.

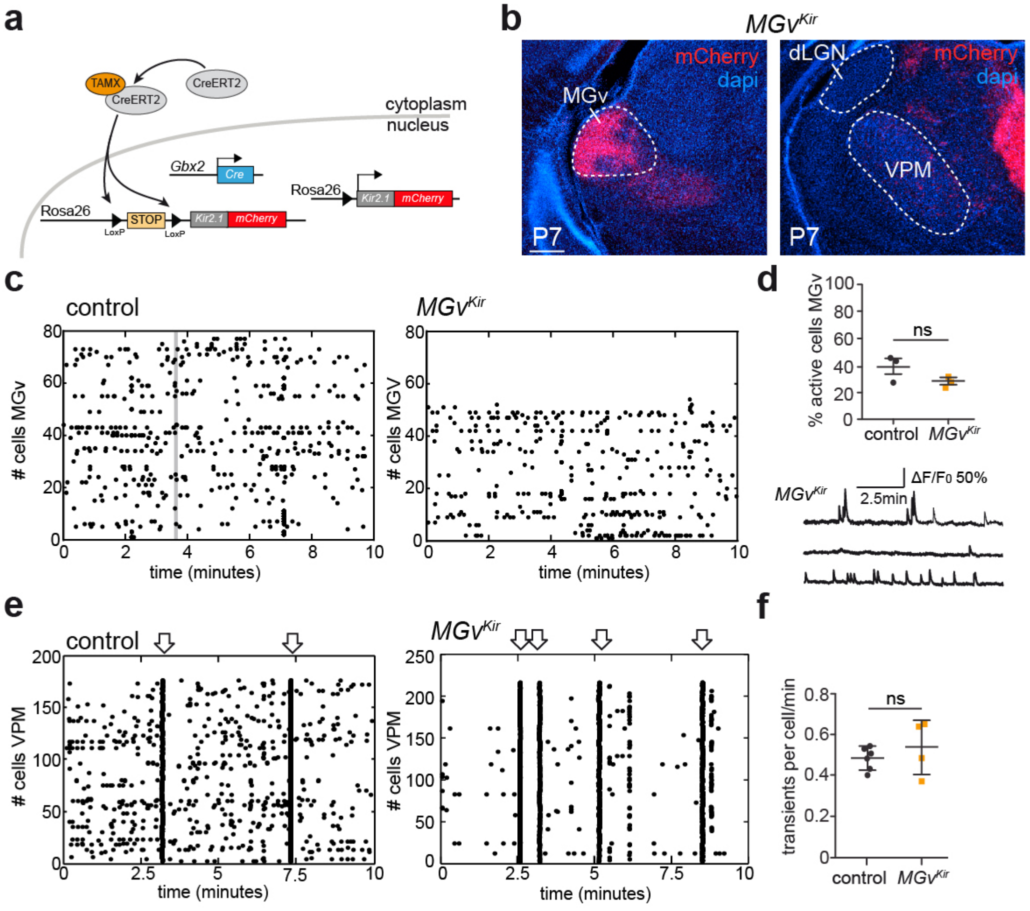

Supplementary Figure 5 Selective targeting of Kir2.1 to the MGv thalamic nucleus in the *MGv*^{Kir} mouse. (**a**) Schema showing the genetic strategy used to selectively overexpress Kir2.1 in MGv neurons. (**b**) Tamoxifen administration at E14.5 specifically targets MGv thalamic neurons in *Gbx2*$^{CreER/+}$*;R26*$^{Kir/+}$ animals. Note the lack of recombination in the somatosensory and visual thalamic nuclei. (**c**) Raster plots of Ca^{2+} activity recorded during 10 minutes in the MGv of control (left panel) and *MGv*^{Kir} expressing (right panel) mice. To quantify the effect of Kir2.1 overexpression, neurons contributing only to the wave (grey line) were removed in the control raster plot for comparison to the *MGv*^{Kir} mouse. In the

present example, 77 out of 273 cells were active in the MGv control and 54 out of 227 in the MGv of the *MGv*^{Kir}. (**d**) Quantification of the percentage of active cells in the MGv nucleus of control (39 ± 6%, excluding the waves; *n* = 3) and *MGv*^{Kir} (28 ± 2%; *n* = 3) mice (upper panel, right); ns, not significant, *P* = 0.3, Mann-Whitney U-test. (**e**) Raster plots showing the onsets of individual cells Ca^{2+} transients at E16.5 in the VPM from control (left panel) and *MGv*^{Kir} (right panel) mice. The higher frequency of waves in the VPM of *MGv*^{Kir} is accompanied by lower inter-wave asynchronous activity. (**f**) Quantification of the average number of Ca^{2+} transients per cell per minute (including asynchronous scatter and wave evoked transients) shows no significant difference between VPM control and VPM *MGv*^{Kir} (control: 0.48 ± 0.02, *n* = 6; *MGv*^{Kir}: 0.54 ± 0.07, *n* = 4; ns, not significant, *P* = 0.61, Mann-Whitney U-test). Graphs represent mean ± SEM.

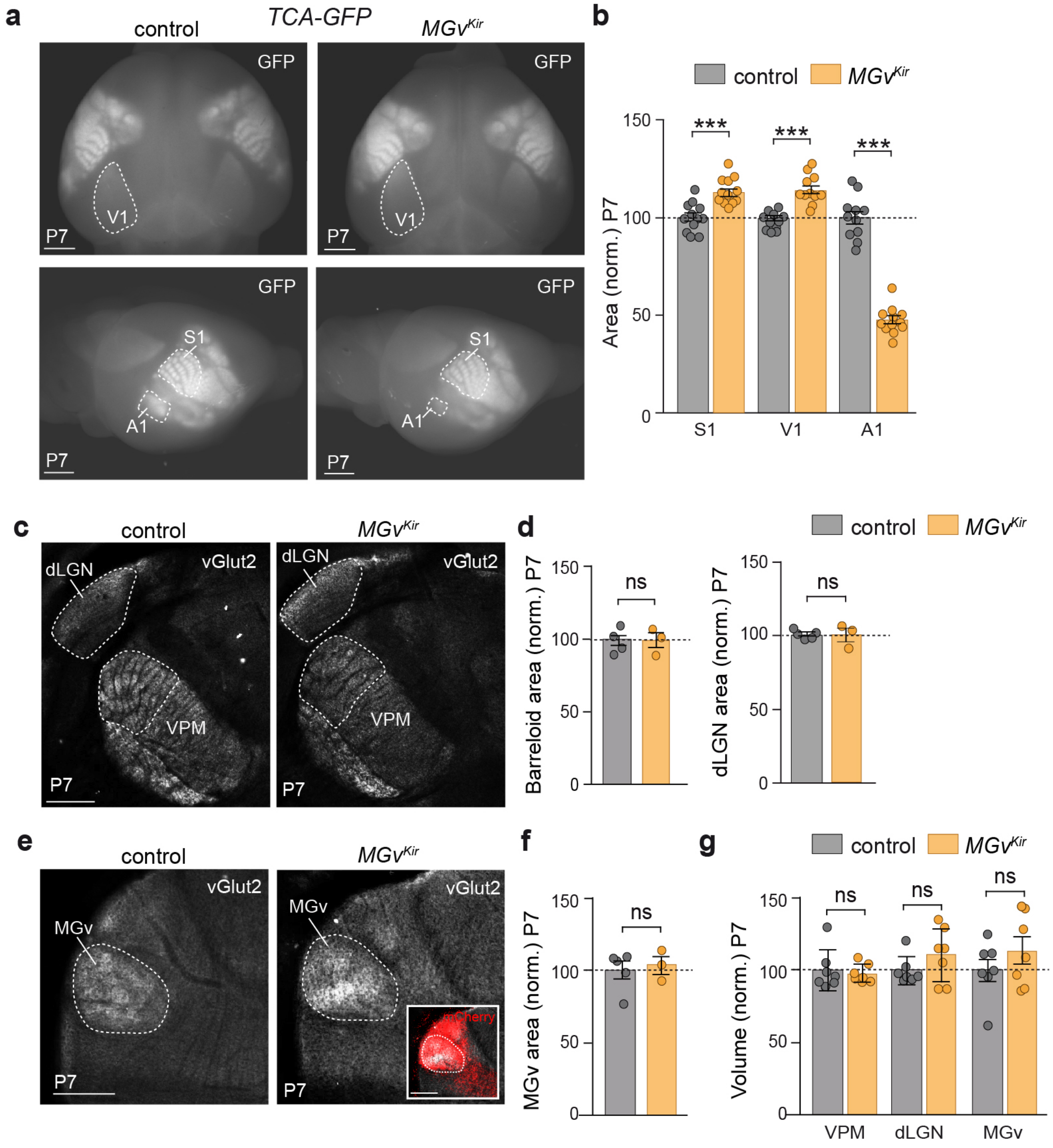

Supplementary Figure 6 Primary cortical areas size modifications in the MGv^{Kir} mouse. (**a**) Labeling of principal sensory cortical areas in control mice (TCA-GFP+) or MGv^{Kir} mice (TCA-GFP+) at P7. **(b)** Quantification of the S1, V1 and A1 areas shown in **a** (S1 area: control: 100 ± 2.19%, n = 11; MGv^{Kir}: 112.3 ± 1.96%, n = 12; ***P < 0.001, Two-tailed Student's t-test. V1 area: control: 100 ± 1.36%, n = 11; MGv^{Kir}: 114.6 ± 2.07%, n = 12; ***P < 0.001, Mann-Whitney U-Test. A1 area: control: 100 ± 3.03%, n = 11; MGv^{Kir}:

48.06 ± 1.99%, *n* = 12; ****P* < 0.001; Two-tailed Student's t-test). (**c**) vGlut2-immunostaining of the VPM and dLGN nuclei of the thalamus in control and MGv^{Kir} mice at P7. (**d**) Quantification of the total barreloid area and dLGN area (barreloid area: control 100 ± 3.43%, *n* = 5, MGv^{Kir}: 99.93 ± 5.37%, *n* = 3, *P*>0.99, Mann-Whitney U-Test. dLGN area: control 100 ± 1.38%, *n* = 5, MGv^{Kir}: 99.44 ± 4.59%, *n* = 3, *P*=0.79, Mann-Whitney U-Test). (**e**) vGlut2-immunostaining of the MGv thalamic nucleus in control and MGv^{Kir} mice at P7. Inset in lower panel shows the recombination in the MGv in MGv^{Kir} mice. (**f**) Quantification of the total MGv area (control 100 ± 6.11%, *n* = 5; MGv^{Kir}: 103.8 ± 6.07%, *n* = 3; *P*>0.99; Mann-Whitney U-Test). (**g**) Quantification of the volumes of VPM, dLGN and MGv (VPM: control 100 ± 5.35%, *n* = 7; MGv^{Kir}: 97.76 ± 2.44%, *n* = 7; *P*=0.71; Two-tailed Student's t-test. dLGN: control 100 ± 3.67%, *n* = 7; MGv^{Kir}: 111.1 ± 7.1%, *n* = 7; *P*=0.19; Two-tailed Student's t-test. MGv: control 100 ± 7.51%, *n* = 7; MGv^{Kir}: 113.9 ± 9.51%, *n* = 7; *P*=0.274; Two-tailed Student's t-test). Graph represent mean ± SEM. Scale bars, 1mm in **a** and 300μm in **c** and **e**.

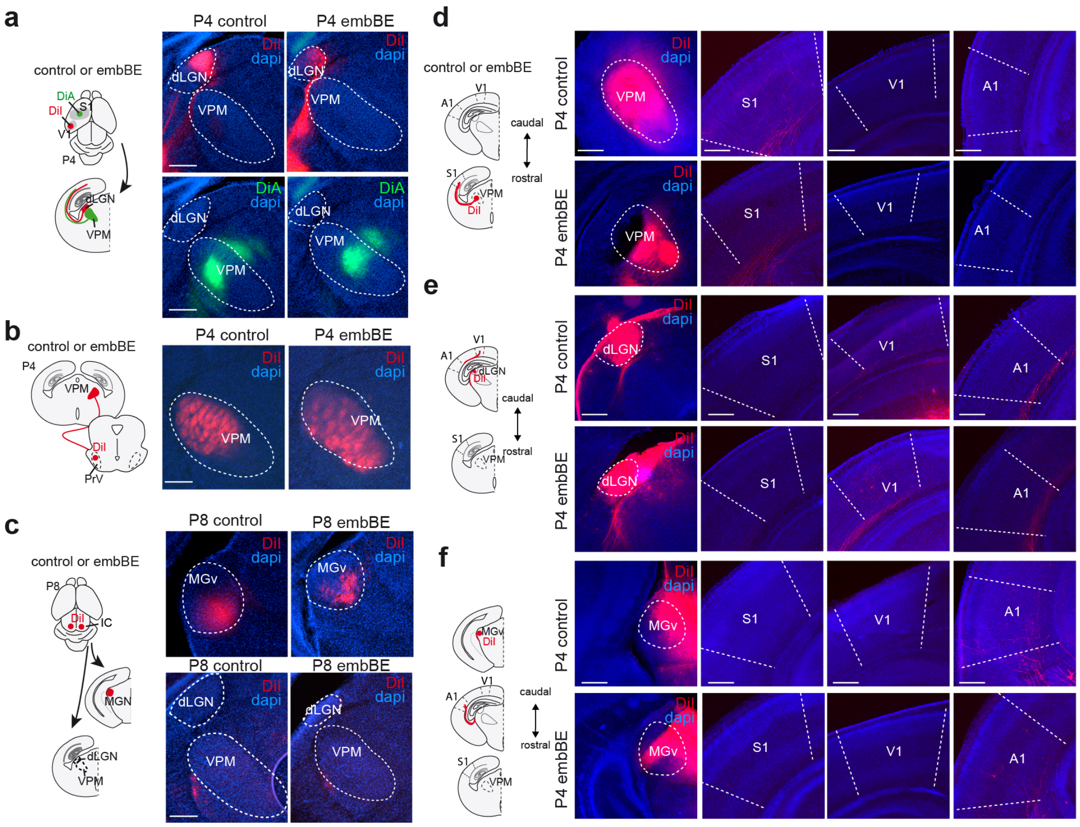

Supplementary Figure 7 Thalamocortical projections and sub-thalamic afferents are not rewired in embBE mice. (**a**) Experimental design to study the effect of embryonic retinal ablation on the topography of TCAs after injection of DiI and DiA crystals into the primary visual (V1) and primary somatosensory (S1) cortices, respectively. Coronal sections show the topography of visual and somatosensory TCAs in control (*n* = 6) and embBE (*n* = 6) mice at P4. (**b**) Experimental design to study the effect of embryonic retinal ablation on the targeting of somatosensory thalamic afferents after injection of DiI into the PrV nucleus of the hindbrain. Coronal sections show PrV axons targeting the VPM thalamic nucleus in both control (*n* = 2) and embBE (*n* = 2) mice at P4. (**c**) Experimental design used to study the effect of removing retinal input embryonically on the targeting of auditory thalamic afferents from the inferior colliculus (IC). Coronal sections show the topographical

connectivity of IC axons into the auditory thalamic nucleus (MGN) in control (*n* = 8) and embBE animals (*n* = 8). (**d-f**) Experimental design to study the effect of embryonic retinal ablation on TCAs after injection of DiI crystals into specific thalamic nuclei at P4 (VPM, **d**; dLGN, **e** and MGv, **f**). Coronal sections show normal targeting of TCAs to S1, V1 or A1 cortices, respectively. Control (*n* = 3) and embBE (*n* = 3) mice. Scale bars, 300μm.

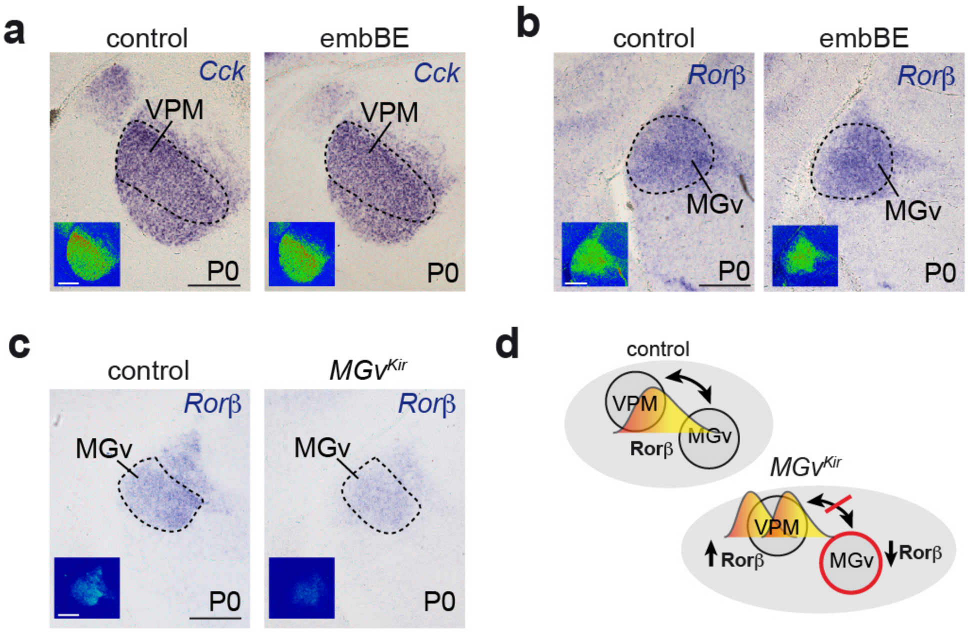

Supplementary Figure 8 Downregulation of *Rorβ* expression in the MGv in the absence of thalamic waves in this nucleus. (**a**) *In situ* hybridization for *Cck* in coronal sections from control and embBE animals at P0. Note that the expression of *Cck* is not altered in the VPM. (**b**) *In situ* hybridization for *Rorβ* in coronal sections from control and embBE animals at P0. Note that the expression of *Rorβ* is not altered in the MGv. (**c**) *Rorβ* mRNA expression in coronal sections from control (n = 5) and MGv^{Kir} (n = 5) animals at P0. Note that *Rorβ* is downregulated in the MGv. (**d**) Schema representing the results found on the changes in *Rorβ* expression in the MGv^{Kir} mouse. Scale bars, 300µm.

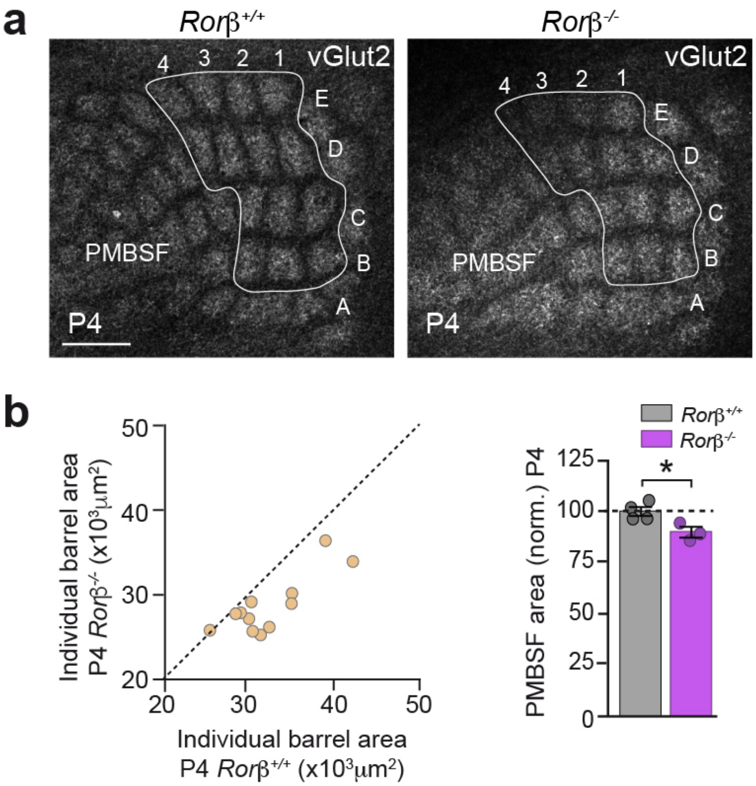

Supplementary Figure 9 Reduced PMBSF in the absence of *Rorβ*. (**a**) vGlut2-immunostaining in tangential sections of the PMBSF at P4 from *Rorβ*$^{+/+}$ (*n* = 4) and *Rorβ*$^{-/-}$ mice (*n* = 3). (**b**) Quantification of the PMBSF area at P4 in *Rorβ*$^{+/+}$ and *Rorβ*$^{-/-}$ mice (*P = 0.02; Two-tailed Student's t-test). Graphs represent mean ± SEM. Scale bars, 300μm

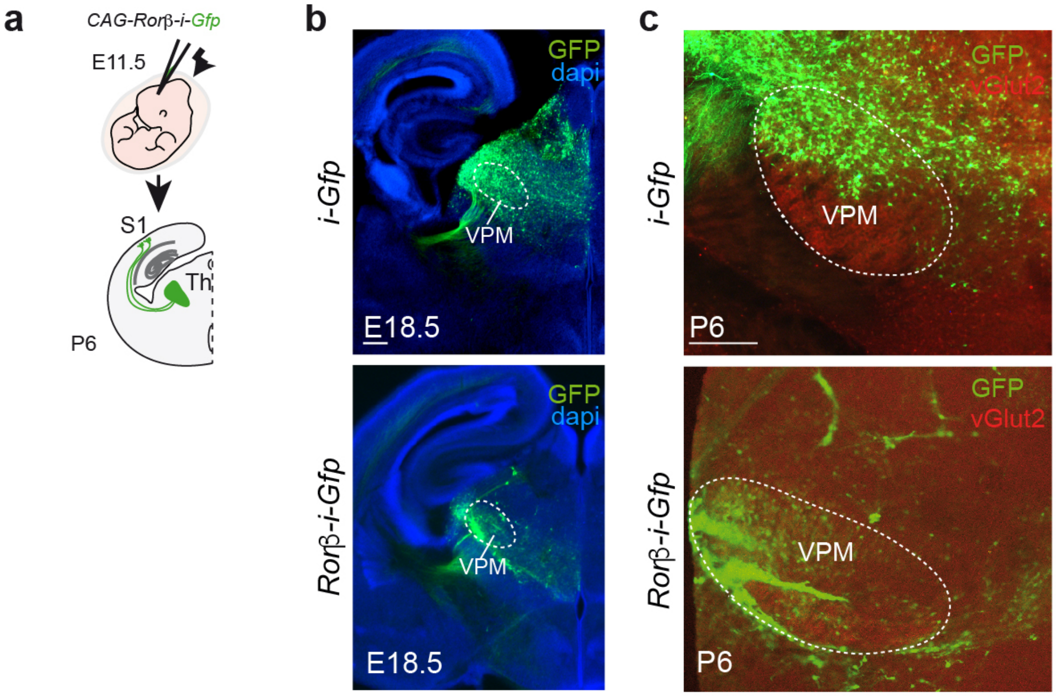

Supplementary Figure 10 Overexpression of *Rorβ* in VPM neurons. (**a**) Scheme of the experimental paradigm used to test the effect of thalamic *Rorβ* gain-of-function on the development of the somatosensory cortex. (**b**) E18.5 coronal sections showing GFP immunostaining (green) and dapi staining (blue) in brains electroporated with a *i-Gfp* or *Rorβ-i-Gfp* constructs at E11.5. (**c**) Coronal sections of P6 brains electroporated with a *i-Gfp* or *Rorβ-i-Gfp* constructs at E11.5 showing vGlut2 and GFP immunostaining in the VPM nucleus. Scale bars, 300μm.
